# On using the dosimetric leaf gap to model the rounded leaf ends in VMAT/RapidArc plans

**DOI:** 10.1120/jacmp.v15i2.4484

**Published:** 2014-03-06

**Authors:** Stanislaw Szpala, Fred Cao, Kirpal Kohli

**Affiliations:** ^1^ Department of Physics BC Cancer Agency Surrey BC Canada

**Keywords:** RapidArc, volumetric‐modulated arc therapy (VMAT), dosimetric leaf gap (DLG), GAFCHROMIC film

## Abstract

Partial transmission through rounded leaf ends of Varian multileaf collimators (MLC) is accounted for with a parameter called the dosimetric leaf gap (DLG). Verification of the value of the DLG is needed when the dose delivery is accompanied by gantry rotation in VMAT plans. We compared the doses measured with GAFCHROMIC film and an ionization chamber to treatment planning system (TPS) calculations to identify the optimum values of the DLG in clinical plans of the whole brain with metastases transferred to a phantom. We noticed the absence of a single value of the DLG that properly models all VMAT plans in our cohort (the optimum DLG varied between 0.93±0.15 mm and 2.2±0.2 mm). The former value is considerably different from the optimum DLG in sliding window plans (about 2.0 mm) that approximate IMRT plans. We further found that a single‐value DLG model cannot accurately reproduce the measured dose profile even of a uniform static slit at a fixed gantry, which is the simplest MLC‐delimited field. The calculation overestimates the measurement in the proximal penumbra, while it underestimates in the distal penumbra. This prompted us to expand the DLG parameter from a plan‐specific number to a mathematical concept of the DLG being a function of the distance in the beam's eye view (BEV) between the dose point and the leaf ends. Such function compensates for the difference between the penumbras in a beam delimited with a rounded leaf MLC and delimited with solid jaws. Utilization of this concept allowed us generating a pair of step‐and‐shoot MLC plans for which we could qualitatively predict the value of the DLG providing best match to ionization chamber measurements. The plan for which the leafs stayed predominantly at positions requiring low values of the DLG (as seen in the profiles of 1D slits) yielded the combined DLG of 1.1±0.2 mm, while the plan with leafs staying at positions requiring larger values of the DLG yielded the DLG 2.4±0.2 mm. Considering the DLG to be a function of the distance (in BEV) between the dose point and the leaf ends allowed us to provide an explanation as to why conventional single‐number DLG is plan‐specific in VMAT plans.

PACS numbers: 87.56.jf, 87.56.nk

## I. INTRODUCTION

Use of rounded leaf ends in multileaf collimators (MLC) in linacs manufactured by Varian (Varian Medical Systems, Palo Alto, CA) requires correcting for effects not present in solid‐jaw delimited fields. In particular, the leaf positions need to be shifted from the nominal positions to account for different geometry when the leafs are not at the central axis, and additional dose in the penumbra due to partial transmission through the rounded ends should be added. The former effect is referred to as the leaf position offset (LPO), and is define as the difference between the optical field size and the nominal leaf position. The LPO is corrected for in the MLC controller software using a table of corrections to the nominal leaf positions depending on the distance from the central axis.^1^, ^2^ The partial transmission of radiation through rounded leaf ends is modeled in Eclipse treatment planning system (TPS) (Varian Medical Systems) through increasing the distance between the opposite leafs, following work of Wang et al.^(3)^ The increase in the field size (measured at the isocenter) along the direction of leaf movement from the field size set by the ends of the leafs is referred to as the dosimetric leaf gap (DLG).^(4)^ The DLG is related to the radiation field offset (RFO), and numerically is twice as large.^(2)^

Rangel and Dunscombe^(5)^ estimated consequences of systematic errors in leaf positioning in dynamic intensity‐modulated radiation therapy (IMRT) plans, which produce a similar effect in matching the calculated to the measured dose distribution as modifying the value of the DLG. They simulated plans in Eclipse, and concluded that 2% error in the PTV dose is introduced when all leafs are opened up by about 0.3 mm in head and neck (H&N) plans and about 0.7 mm in prostate plans.

LoSasso et al.^(6)^ computed the DLG for static fields in Varian MLC (Mark II) from integrals of the dose profiles measured with film along the direction of leaf travel. They deduced the DLG from the line intercept in the plot of the integral of the profile versus MLC gap. They obtained the value of the DLG of 1.7±0.1 mm for a 6 MV photon beam.

Mei et al.^(7)^ employed a variety of methods to find the optimum value of the DLG for use in dynamic IMRT plans for 6 MV photon beams. They inferred the value of the DLG from time‐integrated reading of an ionization chamber placed in the field of a MLC window sliding across the chamber for decreasing values of the width of the window. The authors extended the method to probe variations of the DLG in two dimensions across the field by applying the same methodology to each pixel of an EPID. The authors used another approach to find the dependence of the DLG on the position in one dimension across the field — they analyzed the time plot of the dose rate measured with a diode placed in a sliding window field. They deduced the value of the DLG from the full width at half maximum (FWHM) of the peak, and determined the dependence of the DLG on the location across the field through analysis of the data for diodes of a 1D profiler array. They found the value of the DLG to depend not only on the location along the array, but also on the number of control points governing movement of the MLC leafs, and ranging from about 1.7 mm to 2.5 mm. The dependence of the result on the number of control points suggests that the accuracy of methods utilizing dynamic movement of MLC leafs depends on the uniformity of the velocity of the leafs.

In 2008, Otto^(8)^ introduced volumetric‐modulated arc therapy (VMAT), a.k.a. RapidArc (Varian Medical Systems), where not only the dose was delivered during movement of the gantry, but the dose rate and the gantry speed were modulated. Ling et al.^(9)^ adopted the Picket Fence test for quality assurance (QA) of MLC during dynamic movement in RapidArc. Korreman et al.^(10)^ demonstrated the delivered dose distribution measured with a diode array to be in agreement with the TPS for over 96% of points in the gamma maps calculated at 3% and 3 mm. Masi et al.^(11)^ developed a protocol for dosimetric verification of VMAT, which compares TPS dose distributions to the one measured with film (radiographic and GAFCHROMIC), a diode or an ionization chamber array. Although the authors provided extensive analysis using gamma maps and investigated delivery faults, including linear shift of the leafs and the gantry offset, they did not address the concept of the DLG, which is a part of QA of VMAT.

Van Esch et al.^(12)^ developed a comprehensive set of procedures for implementing RapidArc, from machine QA to TPS validation and patient QA, including validation of the dose distribution in artificial structures created in a phantom. In order to address the correctness of the chosen value of the DLG, the authors analyzed dose profiles measured with GAFCHROMIC film for a MLC pattern set by a 3 mm wide MLC window combined with a 2 cm×2 cm control square. The authors compared these dose profiles with the profiles calculated using Eclipse TPS for the value of the DLG previously determined from ion chamber measurements in the sweeping gap configuration.^(13)^ The actual value of the DLG used in the calculation is not explicitly stated, other than referred to by Chui et al.^(14)^ and a range of numbers published by Van Esch et al.^(13)^

For QA purposes, Bhagwat et al.^(15)^ combined the oscillating movement of an MLC gap with gantry rotation to irradiate a cylindrical target in a quasi‐uniform manner. The authors measured the point dose as a function of the MLC opening and compared the results to a model utilizing a single‐value DLG obtained during commissioning.

In 2011 we reported a preliminary investigation on hw the DLG influences agreement between the computed and the measured (with film) 2D dose distribution in a typical RapidArc plan of the whole brain with metastases.^(16)^ For each value of the DLG, the plan was optimized on the patient CT, transferred to a cubic phantom, calculated using the given value of the DLG, and delivered to the phantom with GAFCHROMIC film embedded in it. The optimum agreement between the TPS and the film data was for the DLG of 1.6 mm.

Kielar et al.^(17)^ observed that the value of the DLG obtained using a moving‐slit test is not appropriate for VMAT plans and leads to as much as 5% discrepancies between the calculated and the measured dose.

In this paper, we compare film and ion chamber measurements to anisotropic analytical algorithm (AAA)^4^, ^18^ calculations in order to identify the optimum DLG in selected plans, and we attempt to provide an explanation of how the DLG influences the dose calculations in VMAT plans delivered using MLCs with rounded leaf ends. We begin with establishing that the point dose measurements are adequate to determine the DLG in VMAT plans. Subsequently, we demonstrate that there is no single value of the DLG that fits all our VMAT plans. By comparing the calculated dose profiles of MLC slits to the corresponding measurements with film, we observe that the calculation overestimates the measured dose in the proximal penumbra, while it underestimates in the distal penumbra. This leads us to consider the optimum DLG not being a plan‐specific quantity, but a function depending primarily on the distance between the point of measurement and the nominal edge of the field in beam's eye view (BEV). Such function may be understood as a way of quantifying the difference between the penumbra in a beam delimited with a rounded leaf MLC and delimited with solid jaws. We provide examples of plans with different values of the single‐value optimum DLG, and explain how the value of the single‐value optimum DLG can be understood based on the fractions of the dose being delivered from specific regions of the penumbra.

## II. Materials and Methods

### A. Planning and dose delivery

We investigated both VMAT and fixed gantry (0�) plans. Within the fixed gantry category, we examined the following types of plans: fixed‐width MLC slits, step‐and‐shoot combinations of fixed‐width MLC slits, and sliding window plans.

We planned and delivered all fields, including both the VMAT and the fixed gantry fields, on a Varian iX linac with Millennium 120 MLC (equipped with 0.5 cm wide leafs near the center and 1 cm wide leafs elsewhere) and controlled through Millennium MLC software, version 7.2. The MLC was calibrated using a standard method recommended by Varian. All fields were 6 MV photon beams, as this energy is commonly chosen in VMAT plans. In all cases, the measurements were normalized to the dose measured in water in reference conditions (1 cGy/MU at SAD 100 cm, 5 cm depth, 10 cm×10 cm) during each session.

The dose distribution in all cases was calculated using AAA version 10.0.28. The smallest allowed dose grid (1 mm) was used in recognition of the impact of the grid size on the calculated dose.(19) The leaf transmission was set to 0.016 for all calculations based on our measurements using an Exradin A19 ionization chamber (0.6 cm3 volume; Standard Imaging, Middleton, WI). The leaf transmission was measured as the ratio of the dose at 5 cm depth in water (at the SSD = 95 cm) between MLC‐blocked beam with totally closed MLC and the leafs junction positioned 7 cm away from the chamber to the dose for the open beam. The chamber was placed approximately at the cax (underneath the nearest leaf to minimize the contribution of the interleaf transmission), and the jaws were set symmetrically to X=Y=20 cm in both measurements. Both the position of the chamber and the field size were chosen arbitrarily (the chosen field size was meant to rpresent typical field sizes used clinically) and may affect the measured value of the leaf transmission slightly. The impact of the value of the leaf transmission on our conclusions is discussed in Discussion section IV below.

#### A.1 VMAT plans

**Figure 1:**
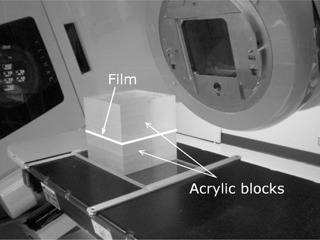
The acrylic‐cube phantom used in measurements of the dose distribution in VMAT plans. The GAFCHROMIC film, placed here in the coronal plane, is sandwiched between two blocks of the phantom.

We use clinical VMAT plans of the whole brain with metastases in the brain, similar to the simultaneous integrated boost described by Hsu et al.,^(20)^ but the metastases were irradiated to the dose of 50 Gy and 40 Gy to the GTV and the PTV, respectively (in 5 fractions), while the remaining part of the brain received the total dose of 20 Gy. The avoidance structures included the spinal cord and the optics (> 25 Gy), with the anterior chamber constrained to 10 Gy. All plans were double (full) arcs, with the collimator set to 30° for one arc and 330° for the other (or 45° and 315°). The plans had been optimized using the Progressive Resolution Optimizer (PRO) (version 10.0.28) in Varian Eclipse TPS. The plans were transferred to CT of a cubic phantom (side = 20.3 cm) made of acrylic (density = 1.18 g / cm^3^) (Fig. 1), and the dose distribution was recalculated with AAA 10.0.28. Following guidelines from the manufacturer, VMAT fluence resolution was set to ‘High’ (0.31 mm). The maximum number of MLC control points (177) was used. One hundred arc calculation segments (the maximum allowed within Eclipse) were use for each arc in order to eliminate the dependence on the number of arc segments. (The terms ‘MLC control points’ and ‘arc calculation segments’ are define in Eclipse Algorithms Reference Guide.^(4)^) The actual phantom was made out of two, approximately equally thick slabs and film was placed in between the slabs. The phantom also had a cavity for ionization chamber measurements. After transferring each clinical plan into the phantom, the isocenter was repositioned so that the film would intersect the center of the investigated planning target volume (PTV) or the chamber would be in the middle of the PTV

#### A.2 Fixed width MLC slits

All fixed‐gantry (0°) fields, including fixed width MLC slits, were also designed using Varian Eclipse, but the positions of the leafs were programmed with Varian MLC Shaper 6.2.

In the fixed width MLC slit plans, all leafs within each bank were opened by half of the investigated slit width, and the leafs in the opposite banks were positioned symmetrically. The jaws were set to X=35 cm (i.e., along the direction of leaf movement) and Y=40 cm. The fields were planned and delivered in a Solid Water phantom. The dose profiles along the direction of the leaf travel were measured with film (see Materials and Methods section B.1 below for details of measurements using film). The film was placed at 5 cm depth, and the SSD was set to 95 cm.

#### A.3 Step‐and‐shoot combinations of fixed width MLC slits

**Figure 2:**
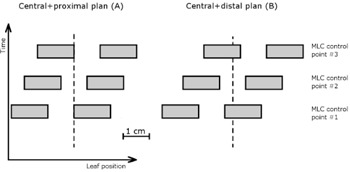
The leaf positions in (a) ‘central+proximal’ and (b) ‘central+distal’ plans in three MLC control points (shown at the top, the center, and the bottom). The leafs move left‐right in this view, and the position of the crosshair is marked with a dashed line. All leaf pairs are displaced by the same amount and, for clarity, only one of the leaf pairs is shown.

Two step‐and‐shoot combinations of fixed width MLC slit fields were planned and delivered in water at the SSD of 95 cm. In each of these two plans, a 1 cm wide uniform MLC slit was positioned at three positions: symmetrically at the central axis(cax), and with positive and negative offset from the cax. The two plans differed by the amount of offset and the number of MUs in each step of the plan. The leaf positions with respect to the cross hair are shown in Fig. 2 for the representative MLC control points in each plan. Only one leaf pair is shown, as the others were set identically. In plan A (later referred to as the ‘central+proximal’ plan), the slit offset from the cax was set to ±0.5 cm, and 71 MU, 114 MU, and 71 MU were delivered at the offset of −0.5 cm, no offset, and 0.5 cm offset, respectively. In plan B (later referred to as the ‘central+distal’ plan), the slit offset was set to ±0.8 cm, and 321 MU, 912 MU, and 321 MU were delivered at the offset of −0.8 cm, no offset, and 0.8 cm offset, respectively. The MUs were chosen arbitrarily in both plans. The jaws were set to X=35 cm and Y=40 cm.

The point dose in these step‐and‐shoot plans was measured with an ionization chamber (see Materials and Methods section B.2). The chamber was placed at 5 cm depth, but not directly on the cax. It was shifted 0.25 cm (one‐half of the width of an MLC leaf) perpendicularly to the direction of leaf movement to minimize the contribution of the interleaf transmission. To minimize the influence of the setup uncertainty on the readings, the position of the ionization chamber was adjusted along the direction of the leaf movement so that the dose reading from the part of the plan delivered under the negative offset of the chamber with respect to the cax would match the reading during the positive offset.

#### A.4 Sliding window plans

In the sliding window plans, the field was collimated primarily with the MLC to form a slit of required width, and all leafs were set identically in each bank. Five equally spaced MLC control points were programmed instead of only two required to define the sliding window movement. The choice of five control points was made following work of Mei et al.,^(7)^ where the authors observed a considerable discrepancy between the measurement and the calculation when using only two control points. The leaf trajectory and the jaw openings were chosen similarly to Bhagwat et al.^(15)^ (i.e., the X jaws were set to (symmetric) 12 cm and the center of the MLC window coincided with the edge of one of the X jaws at the beginning and the end of the movement of the leafs). The Y jaws were set to symmetrical 20 cm. The fields were delivered in water at the SSD=95 cm, at the gantry and the collimator angles equal zero. Four hundred (400) MUs were delivered in each case at the dose rate of 600 MUs/min. The point dose in the sliding window plans was measured with an ionization chamber positioned at the depth of 5 cm.

### B. Measurements of the dose

We combined ionization chamber data with film data to take advantage of dosimetric accuracy of ionization chambers and spatial resolution of film. We did not attempt using ionization chamber arrays because the spacing between the detectors (7.6 mm for MatrixX Evolution; IBA Dosimetry GmbH, Schwarzenbruck, Germany) is too large compared to the steep dose gradients encountered in this project.

#### B.1 Film

**Figure 3:**
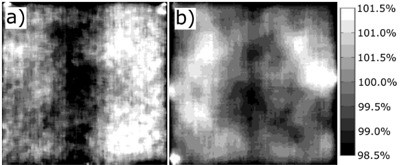
Measured dose distribution in 5 cm×5 cm squares of EBT1 film irradiated uniformly to the dose of (a) 200 cGy and (b) 900 cGy using 6 MV photon beams. The distributions are normalized to the average dose in each square.

**Figure 4:**
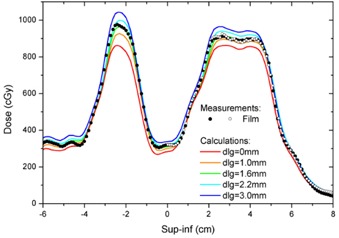
The calculated and measured with film superior‐inferior profiles across two PTVs in the VMAT plan analyzed in the Results section A.1. The calculated dose (color lines) in the center of each PTV increases with an increase of the DLG and matches the measured profile (black circles) for the DLG of about 1.6 mm (green line). Repeated film measurements are plotted as open circles.

For measurements with film, in both VMAT and in fixed gantry plans, GAFCHROMIC EBT1 film (International Specialty Products, Wayne, NJ, currently Ashland, KY) was positioned between the slabs of the phantom. We chose to measure the dose profile using film due to its excellent spatial resolution, and GAFCHROMIC EBT film was selected due to having almost no energy dependence. EBT1 film was chosen over EBT2 due to its better uniformity. Typical response of EBT1 film to irradiation with 200 cGy and 900 cGy at the depth of 5 cm using 10 cm×10 cm 6 MV photon beams is shown in Fig. 3 for 5 cm×5 cm squares. The distributions are normalized to the average dose in each square piece. In spite of uniform irradiation of the film, regions of low response and high response are visible, and the relative readings vary from about 98.5% to about 101.5% of the average dose received by each piece. This relative uncertainty of 1.5% from the average dose does not depend on the dose received by the film within the investigated range of doses. This uncertainty has little effect on the conversion from the pixel intensity to cGy during calibration of the films, because 5 cm squares are large enough to average‐out nonuniformity in response of the film to radiation. Measuring a point dose, on the other hand, is affected by nonuniform response of the film, and we subsequently assumed 1.5% uncertainty of reading the dose using EBT1 film. This value is comparable to the uncertainties reported by van Battum et al.^(21)^ for EBT1 films. For calibration purposes, additional film pieces were irradiated in reference conditions (5 cm depth in water at SAD 100 cm with the field size of 10 cm×10 cm) together with the investigated film. For the VMAT plans, the film calibration was performed at the doses of 0, 300, 600, 900, and 1100 cGy, and fitted with a 4th degree polynomial. For the fixed gantry plans, the film was calibrated at 0, 20, 50, 100,

200, 300, and 350 cGy and cubic‐polynomial interpolation was used in between. All films were scanned in the transmission mode of the Epson Perfection V700 photo scanner (US Epson, Long Beach, CA). Only the red channel was analyzed, all exposure corrections were turned off, and the resolution was set to 72 dpi for VMAT plans and 150 dpi in other cases. Although a lower resolution was used for VMAT plans, 72 dpi corresponds to 0.35 mm spacing between pixels, which is more accurate than 1 mm grid used in Eclipse dose distributions (the best available), to which the film data were compared. Moreover, inspection of the dose gradients encountered in our plans (Fig. 4) demonstrates that the curves were not undersampled at the resolution of 72 dpi. The investigated films were placed on the scanner glass in the same orientation as the calibration films because the sensitivity of GAFCHROMIC film is known to depend on the film orientation on the scanner glass.^(22)^ To compensate for the dependence of the readings on the location at the scanner glass and similarly to the protocol developed by Menegotti,^(23)^ a position‐dependent multiplicative correction function (an inverted‐Gaussian along the light source bar direction of the scanner and no correction across the light source bar) was applied to the pixel intensities. In addition, the pieces of film for measuring the dose profiles were placed such that the investigated profile was lined up along the direction of movement of the light source (where the readings do not depend on the position on the scanner glass). Care was taken to scan the investigated and the calibration film pieces together to eliminate time‐dependent self‐developing of film following irradiation.^(24)^ The films were scanned only once to eliminate additional film darkening by the light of the scanner. The pixel intensity was converted to cGy using in‐house‐designed software. No dose normalization was employed in order to compare the absolute calculated and the measured doses. The registration of the dose distributions measured with film in VMAT plans to the calculated dose distributions was based on marks on the film along the light crosshair. The line profiles in fixed gantry MLC slit beams were manually registered to the calculated profiles to match the cax obtained as the midpoint between 50% doses in the penumbras. Each pixel of the profile was calculated as the average of all pixels up to 7.5 mm on both sides across the profile. Such averaging was performed for consistency with Eclipse, where inter‐ and intraleaf transmission is modeled with a single average number, as well as to minimize the error caused by film nonuniformity.

#### B.2 Ionization chamber

We use Exradin A16 ionization chamber (Standard Imaging, Middleton, WI) for measurements of the point dose, and we chose this model due to its small size (2.4 mm inner diameter of the collecting volume). We converted the readings to cGy following exposure to a known dose in reference conditions in water. To account for the effective collecting volume of the chamber, we averaged the dose in a structure in Eclipse designed to mimic the chamber. Following the work of Tessier et al.,^(25)^ we use the collecting volume of the chamber to contour this structure in Eclipse. We did not compensate for the effective point of measurement because it is only about 0.3 mm (upstream) for the A16 chamber.^(25)^

## III. RESULTS

### A. VMAT plans

#### A.1 Film measurements

The superior‐inferior profile measured with film passing through two PTVs (about 1 cm in diameter and about 2 cm in diameter) of a VMAT plan transferred to the cube phantom is shown in Fig. 4 (solid circles), together with the corresponding profiles calculated in Eclipse for various values of the DLG ranging from 0 to 3 mm (solid lines). The repeated film measurements (open circles) for the larger PTV are also shown to demonstrate reproducibility. The original clinical plan was optimized using the DLG = 1.6 mm. The AAA profiles are influenced by the value of the DLG used in the calculation. The maximum calculated dose increases with an increase of the DLG, and this is a consequence of modeling the transmission of radiation through rounded leafs by introducing an additive component to the fluence. The effect is stronger for the smaller PTV, but is also noticeable for the larger PTV.

Although the optimum value of the DLG may be identified by choosing the AAA profile which fits the measured data best, we chose not to do it here because of a fairly large error (about ±1.5%) of film measurements, which affects accuracy of determining the optimum DLG. Upon noticing that the point dose in the middle of a PTV is affected by the DLG, we repeated the measurements using an ionization chamber, which is a more accurate dosimeter than film (see the next section).

#### A.2 Ionization chamber measurements

**Figure 5:**
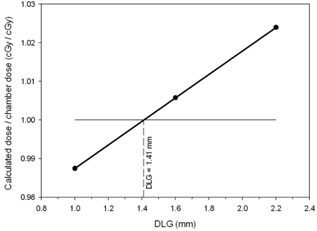
The ratio of the calculated to the measured (with an ionization chamber) dose as a function of the DLG. The ratio equal one indicates a perfect match, which is at the DLG of 1.41 mm.

We measured the point dose with an ionization chamber in VMAT plans of five patients for the total of seven PTVs (two PTVs from within a single plan were analyzed in some cases). The diameter of the PTVs analyzed with an ionization chamber ranged between 1.6 cm and 2.5 cm. We only did measurements for those PTVs where the calculated dose profiles were fairly flat near the center of the PTV. The ratios of the calculated dose (average in the collecting volume structure mimicking the chamber) for the DLG = 1.0, 1.6, and 2.2 mm to the measured dose in one of these PTVs are shown in Fig. 5 (solid circles interconnected with thick solid line). The optimum value of the DLG was identified for which the calculated dose was equal the measured dose (i.e., at the intersection of the thick solid line with the unity line). Here, the optimum DLG is 1.41 mm.

**Figure 6:**
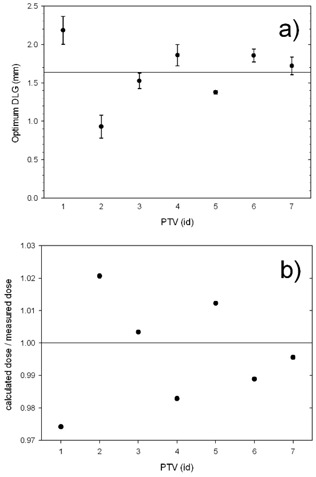
The optimum DLG (a) found in VMAT plans of the brain with metastases. The horizontal axisdenotes the sequential number of the PTV. The values of the optimum DLG vary from 0.93±0.15 mm and 2.2±0.2 mm. The average value of the DLG for this dataset is plotted as a line (DLG=1.64 mm), but there is no single value of the DLG that adequately describes the entire cohort. The ratio (b) of the dose calculated using the DLG=1.64 mm (the average DLG) to the value measured with an ionization chamber.

The optimum DLG for all analyzed PTVs is shown in Fig. 6(a). Please note that the abscissa denotes the sequential number only, and one could reorder the data points without affecting the meaning of the graph. Each data point is an average of four measurements (with the exception of PTV #3 and #7, where only three measurements were collected). The measurements were repeated on separate days (with the setup redone each time) in order to take into account the setup uncertainty. The MLCs were initialized at the beginning of each session. The repeated measurements were collected over the span of several months. The error bars for each PTV denote the standard deviation calculated for the series of repeated measurement. The optimum DLG in the analyzed set is between 0.93±0.15 mm and 2.2±0.2 mm. These minimum and maximum values differ by about nine error bars, implying the absence of a single value of the DLG fitting all cases. We also checked the magnitude of the discrepancy between the calculated and the measured point dose upon using the average value of the DLG in the calculation, which is 1.64 mm. The ratio of the dose calculated using the DLG = 1.64 mm to the value derived from the measurement using the ionization chamber is plotted in Fig. 6(b) for the same set of PTVs. In two out of seven PTVs, the discrepancy is between 2% and 3%, while in five out of seven PTVs the discrepancy exceeds 1%.

#### A.3 Leaf movement patterns

**Figure 7:**
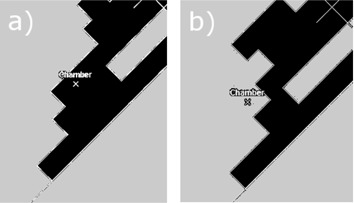
Typical MLC apertures in BEV found in VMAT plans plotted on top of the dose point located in the middle of the PTV. Not only the dose from inside of the geometrical beam aperture (a) contributes to the dose at the dose point, but also the dose from underneath the geometrical shadow of the leafs (b) (i.e., the dose from the distal penumbra).

In the VMAT plans that we investigated, the leaf movement consisted of patterns considerably different from those found in typical dynamic IMRT plans (H&N, breast or prostate). In the dynamic IMRT plans, the leafs typically move quasi‐collectively as a slit travelling across the entire PTV, with only some time‐dependent variations of the spacing between the opposite leafs. In VMAT plans, on the other hand, the MLC aperture changes nonstop while partially or completely crossing over the PTV multiple times. At some MLC control points, a dose point may reside in the beam penumbra. The contribution to the dose from the penumbra may be from either the inside of the beam aperture or from under the geometrical shadow of the leafs. Examples of BEV of the MLC openings in a VMAT plan at two representative MLC control points are shown in Fig. 7, one for the chamber being inside the geometrical outline of the beam and the other for the chamber located under the geometrical shadow of the leafs. Consequences of the pattern of leaf movement will be discussed in the subsequent sections.

### B. Fixed gantry plans

#### B.1 Profiles of fixed width MLC slits

**Figure 8:**
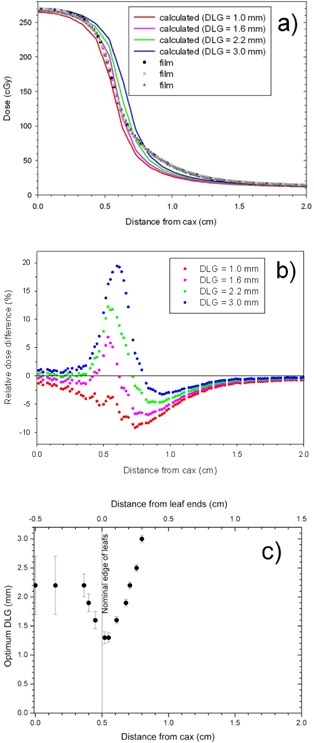
(a) The film (three separate pieces of film irradiated in the same way) and the calculated profiles of 1 cm wide MLC slit in a flat phantom. The profile is symmetric and only the right side from the cax is shown. The residual plots (b) of the calculated and the measured profiles plotted in (a). The DLG (c) may be define at each point of the profile as the value for which the calculation matches the measurement. The top axisis shifted from the bottom axisby 0.5 cm, and indicates the distance measured from the nominal (geometrical) ends of the leafs. The positive values denote positions under the geometrical shadow of the leafs.

Recognizing complexity of the leaf movement accompanied by rotation of the gantry in VMAT plans, we attempted to identify values of the optimum DLG in simpler cases, such as static fields delimited with MLC under fixed gantry condition. The measured profile of a 1 cm wide MLC slit in a Solid Water flat phantom (6 MV, 100 cm SAD, depth = 5 cm, jaws almost fully open at X=35 cm and Y=40 cm), and the corresponding profiles calculated for the DLG between 1.0 mm and 3.0 mm are shown in Fig. 8(a). Only one side of the (symmetrical) profiles is shown. The measured profile (symbols) consists of three datasets obtained from three pieces of film irradiated separately under identical conditions. Variations of the dose in the penumbra region measured using separate pieces of film are smaller than the variations in the AAA profiles plotted for various values of the DLG. It turns out there is no value of the DLG for which there is a good match between the calculated and the measured profile for all distances from the cax. Visually the best match is obtained for the DLG=1.6 mm but, even in this case, the calculation overestimates the measurements in the proximal penumbra (between 0.45 cm and 0.7 cm from the cax) and simultaneously underestimates in the distal penumbra (beyond about 0.75 cm from the cax). The problem is evident even more clearly when the beam profiles are replotted as the residual plots (the difference between the AAA and the film profiles after normalizing to the dose measured with film at the cax) (Fig. 8(b). The residual value of zero indicates the best match at the given distance from the cax. At 0.5 cm away from the cax (proximal penumbra), the optimum match would be for the DLG of about 1.3 mm (the value interpolated between the plotted data for the DLG of 1.0 and 1.6 mm, but at 0.8 cm (distal penumbra) the optimum DLG would be about 3.0 mm. These values of the DLG required for the optimum match between the measurement and the calculation are significantly different.

Although a better match between the measurements and the calculation could be obtained by increasing the leaf transmission in AAA, we feel this is not an appropriate strategy here. After all, increasing the leaf transmission would substantially reduce accuracy beyond the penumbra, which clinically would lead to inaccurate calculation of the dose to critical organs.

Even though there is no single value of the DLG that provides a reasonable match between the calculated and the measured data simultaneously in the entire profile, nor even at the entire penumbra, the optimum DLG can be computed individually for each point in the profile as a function of the distance from the cax. This is roughly equivalent to a scenario where the width of the MLC slit used in the calculations is adjusted independently for each distance from the cax. Such a scenario is a concept that cannot be implemented without modifying the TPS. In Fig. 8(c) we plotted the optimum DLG as a function of the distance from the cax. For each value of the distance from the cax, the optimum DLG was obtained as the interpolated value for which the AAA calculation matches the measurement (plotted in Fig. 8(a)). The error bars were calculated to account for the uncertainty of measuring the dose using film in the following way. We repeated the interpolation after changing the film dose by 1.5% (the approximate error of the dose measured with EBT1 film), and computed the difference between this new value of the DLG and the value for the as‐measured film dose. This difference represents the error bars. The near‐cax value of the optimum DLG carries large uncertainty because the cax is relatively far from the penumbra for 1 cm wide MLC slit and the DLG influences primarily the penumbra region of the profile. Increasing the distance from the cax leads to a significant reduction of the optimum DLG in the proximal penumbra, with the minimum value of the DLG of 1.3 mm around 0.55 cm distance from the cax (i.e., near the nominal beam width). Further increase of the distance from the cax leads to a gradual increase of the optimum DLG. At around 0.8 cm (i.e., 0.3 cm from the nominal beam edge), the optimum value of the DLG reaches a value of 3.0 mm. As mentioned earlier, we did not adjust the leaf transmission, which would improve the agreement around 1 cm from the nominal edge of the beam. The top horizontal axisis also marked in Fig. 8(c). It is shifted from the distance from the cax by 0.5 cm, and denotes the distance measured from the beam edge (i.e., from the leaf ends).

**Figure 9:**
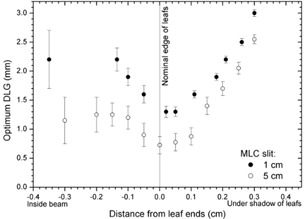
The optimum DLG for 1 cm and 5 cm MLC slits in a flat phantom as a function of the distance from the nominal edge of the leafs.

We observed similar phenomena for fields with different sizes of the MLC slit. The dependence of the optimum DLG versus distance from the beam edge is plotted in Fig. 9 for two widths of the MLC slit: 1 cm and 5 cm. The data for the 1 cm MLC slit were replotted from Fig. 8(c), and the data for the 5 cm slit were obtained from the film profile of the 5 cm wide MLC slit and analyzed in a similar manner as for the 1 cm MLC slit. As for the 1 cm MLC slit, the optimum DLG depends on the distance from the beam edge also for the 5 cm slit. In both cases, the optimum DLG reaches the minimum value around the nominal beam edge. The values of the optimum DLG are considerably smaller though for the 5 cm MLC slit than for the 1 cm MLC slit, with the smallest value of only 0.75±0.15 mm.

#### B.2 Combination plans

It is not practical to draw conclusions applicable to VMAT plans based only on the data presented in Fig. 9, because these data represent merely a pair of identical MLC control points, while VMAT plans consist of a large number of MLC control points. In order to demonstrate the impact of the dependence of the optimum DLG function on the distance from the beam edge, we created a simple pair of step‐and‐shoot plans, each with a different amount of the contribution of the distal and of the proximal penumbra to the point dose at the cax. To separate the MLC‐related effects from the effects associated with the beam direction, we kept the gantry at 0°. The effects related to gantry rotation are not investigated here. In the first plan (plan A, ‘central+proximal’ plan described in the Material and Methods section A.2), other than the on‐axis MLC control point, it is mostly the proximal, not the distal, penumbra that contributes to the dose at the cax, while in the second plan (plan B, ‘central+distal’ plan) it is mostly the distal penumbra. The purpose of the zero‐offset MLC control point in both plans is to have the plans mimic typical VMAT plans, for which the central region of the MLC slit contributes to the dose. Nevertheless, these two plans were not designed to closely resemble actual clinical plans, but to have different contribution to the dose at the cax from the proximal and the distal penumbra.

**Figure 10:**
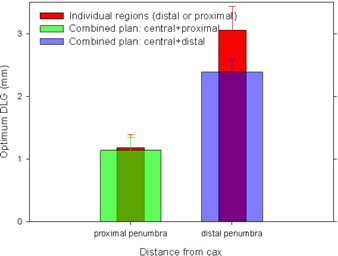
The optimum DLG in ‘central+distal’ and ‘central+proximal’ plans, together with the optimum DLG obtained for the distal and the proximal MLC control points (i.e., the critical components in the combination plans).

For each plan, the dose distribution was calculated for various values of the DLG. For each combination plan and for each MLC control point, the optimum DLG was interpolated for the best match between the calculation and the measurement (in a similar manner as described in the Results section A.2). The interpolated optimum DLG is plotted in Fig. 10. The error bars were obtained similarly as described in Results section A.2 above — the chamber structure was shifted left by 1 mm in the plan, the optimum DLG was reinterpolated, and the difference between the two values of the DLG is interpreted as the uncertainty of the value of the DLG. Only shift to the left was considered because of the symmetry of the plan and only minimal, if any, dose gradients in other directions. The optimum DLG of the MLC proximal control points (MLC‐slit offset from the cax by −0.5 cm or 0.5 cm) is much smaller than for the distal control points (MLC‐slit offset from the cax of −0.8 cm or 0.8 cm), 1.2±0.2 mm and 3.1±0.4 mm, respectively. This is similar to the optimum values of the DLG found in the dose profile of a single 1 cm wide MLC slit (Fig. 8(c)), where the optimum DLG is about 1.2 mm at 0.5 cm from the cax and about 3.0 cm at 0.8 cm from the cax, except it was measured with an ionization chamber instead of film.

The optimum DLG in the combination plans (central+proximal and central+distal) differ significantly: 1.1±0.2 mm and 2.4±0.2 mm, respectively (Fig. 10). The difference is well beyond the uncertainty of the measurements (over six times), and demonstrates that changing the amount of contribution to the dose between the proximal and the distal penumbra affects the optimum value of the DLG.

#### B.3 Sliding window plans

**Figure 11:**
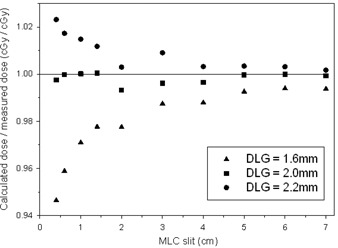
The ratio of the calculated to the measured dose in the sliding window plans as a function of the width of the MLC slit for various values of the DLG. The ratio is approximately one for the DLG=2.0 mm indicating a good match of the calculations to the measurements.

In order to further demonstrate the dependence of the optimum DLG on the pattern of movement of the MLC leafs, we compared the ion chamber readings in sliding window tests to the corresponding AAA calculation for various values of the DLG. The ratio of the calculated to the measured dose for various widths of the MLC opening is plotted in Fig. 11. Like in the previously analyzed examples, increasing the value of the DLG used in the calculation increases the AAA dose and, consequently, increases the ratio of the calculated to the measured dose. The sensitivity of the ratio to changes in the DLG decreases with an increase of the width of the MLC slit. At slits over 4 cm wide, the ratio of the calculated to the measured dose changes less than 1% within the investigated range of the DLG and, hence, the value of the DLG becomes clinically irrelevant. The interesting fact is that a single value of the DLG of 2.0 mm can be use as the optimum value independently on the width of the MLC slit in order to obtain agreement within better than 0.7%. This is a consequence of the dose integration by the ionization chamber over the entire beam profile when the sliding window moves over the chamber whereby the regions of smaller optimum DLG compensate the regions of larger optimum DLG. The independence of the DLG on the MLC opening in the sliding window plans differs from our findings for VMAT plans in the cohort, where the optimum DLG was plan dependent.

## IV. DISCUSSION

One may argue that the DLG and the penumbra effects are not important in VMAT plans because the majority of the dose is delivered from the central portion of the beam. This is not the case of the plans that we investigated, where the value of the DLG significantly influences the planned dose distribution. This was demonstrated in Fig. 4 for a plan of the whole brain with metastases, where variations of the DLG affect the agreement between the calculated and the measured dose, especially around the maximum dose. Moreover, choosing the wrong value of the DLG in this plan translates to an error in the dose, which magnitude is different in the two PTVs. Although we are unable to provide a quantitative prediction of the value of the DLG that should be use, our data demonstrate that such value is plan‐dependent for small PTVs (Fig. 6).

The value of the leaf transmission does not significantly affect the dose profiles in the vicinity of the penumbra. As shown in Fig. 8(a), at the distance of 2.0 cm from the cax (i.e., 1.5 cm from the nominal beam edge), the dose is considerably higher (about three times higher) than the value of the leaf transmission. Most of our observations pertain to the effects related to the dose in the beam penumbra, and consequently we did not further investigate the influence of the value of the leaf transmission on the dose in the high‐dose regions in VMAT plans. Nevertheless, the value of the leaf transmission may affect the dose in the low‐dose regions of VMAT plans, but we did not investigate it.

Allowing the DLG to vary may appear similar to modifying the table of the LPO (as define by Vial et al.^(2)^), but the LPO table is a purely geometrical concept involving only a totally opaque single leaf or a leaf bank (not a pair of opposing leafs), while our data in Fig. 9 demonstrate that the DLG depends on the size of the MLC slit. Such effect cannot be explained on geometrical grounds alone. Change of the DLG with the size of the MLC slit is probably caused by variation in the amount of radiation scattered from the opposite leaf bank upon changing the size of the MLC slit. The “sign” of the effect observed in our data in Fig. 9 is consistent with this explanation: increasing the separation between the opposite leafs reduces the chance of the scattered radiation reaching the dose point under the leaf because, at MeV energies, the photons scatter in the direction close to the direction of the incident photons. This reduction of the dose upon moving the opposing leafs further away may be modeled by decreasing the DLG, which is demonstrated in Fig. 8(a) at the distance from the cax of about 0.5 cm (i.e., near the nominal leaf‐end positions). Because the DLG depends not only on the distance from the middle of the penumbra but also on the position of the opposite leaf, variations in DLG cannot be included in the table of LPO without adding the second parameter (the width of the slit between the opposite leafs or the position of the opposite leaf).

Although we do not provide precise interpretation how the DLG (and the RFO) depends on the distance from the middle of the penumbra, introduction of this function allows more accurate modeling of the difference in the shape of the penumbra between a beam delimited with MLC and a beam delimited with solid jaws. In Eclipse TPS, only solid‐jaws beam profiles are measured during commissioning, and the beam profiles for beams delimited with MLC are modeled simply by shifting the leaf positions without any other modifications to the shape of the beam profiles. Unlike with solid jaws with square endings aligned along the beam divergence at any jaw position, the shape of the beam penumbra in MLC‐delimited fields is influenced by partial transmission through the leaf endings. Moreover, the amount of the radiation transmitted through the rounded leaf ends depends on the distance from the leaf ending, as the beam travels through longer amount of material of the leaf as the distance (under the leaf) from the leaf ending increases. Consequently, the dose correction (modeled using the DLG or the RFO) added to the dose calculated on geometrical grounds assuming totally opaque leafs depends on the distance from the leaf endings.

Extending the concept of the DLG from a single‐value parameter to a function of the distance (in BEV) from the leaf ending to the point where the dose is measured is helpful in understanding why the conventional single‐value DLG is plan‐dependent. For each MLC control point, an optimum DLG can be identified based on this distance. Because this distance can be different for different MLC control points, in general one cannot get a single value of the DLG that provides exact agreement between the measurement and the calculation for all control points simultaneously. Nevertheless, summation of the point dose from all control points may be performed, and we adjusted the DLG to match the total Eclipse point dose to the total measured dose. In general, the optimum DLG obtained with this procedure may depend on the choice of the dose point, especially if the dose point is not in the high‐dose region. We did not investigate here how the optimum DLG depends on the location of the dose point.

The optimum value of the DLG in the plan (at a given point as described above) varies depending on the amount of dose delivered from various MLC control points, each characterized by a given value of the DLG based on the distance between the dose point and the leaf ending (in BEV). As we showed in the Results section A.3, for some MLC control points in VMAT plans, the dose point falls near the edge of the geometrical shadow of the leafs, while for others, it is under the leafs but further from the edge and, based on the data plotted in Fig. 9, the optimum DLG will be higher in the latter case. It is our understanding that different values of the optimum DLG were needed in our VMAT plans because of plan‐dependent amount of time the dose point resides near the leaf ending in BEV or away from it.

We demonstrated that the DLG is plan‐dependent in VMAT plans. We believe it is best to obtain the optimum DLG by measuring the point dose in the middle of the PTV in the verification plan in a phantom, and identify the optimum value by comparing the measured dose to the corresponding calculated one for various values of the DLG, followed by recalculation of the dose distribution using the optimum DLG. We cannot state a numerical error introduced to the dose upon using an incorrect value of the DLG in general, but for the set of PTVs analyzed in this work, the error exceeds 2% upon setting the DLG to 1.64 mm for two out of seven PTVs (and exceeds 1% for five out of seven PTVs) (see Results section A.2). Such errors are meaningful when related, for example, to the TG142‐recommended tolerance of 1% of the annual calibration of cGy/MU for linacs used to deliver IMRT plans.^(26)^

We define the optimum single‐value DLG such that the calculated dose equals the measured dose, implying that all uncertainties in the calculation model are due to the choice of the DLG. Such implication is not true, because there are other parameters in the TPS that affect the calculated dose distribution. In particular, the profiles of beams delimited with MLC are derived from the open‐beam profiles. Consequently, inaccuracy of the open‐beam profiles, which could be caused by the dose integration in the detector during collection of the profiles, will affect the calculated dose distributions in plans utilizing MLC.

We think improvements in agreement between the calculated and the actual dose distribution in VMAT plans can be achieved through better modeling of the beam profiles of rounded leaf ends MLC, not merely through opening the leafs in the TPS by a few millimeters. Because the beam partially penetrates the material of the (rounded) leaf ends in the amount dependent on the distance from the physical edge of the leaf, while in the first approximation the beam does not penetrate solid jaws, one should not expect the beam profile of a MLC‐delimited field could be derived from the beam profile of a beam collimated with jaws simply through shifting the beam profile. Even when considering partial penetration of the jaw material (due to the nonpoint size of the beam at the target of the linac), one should not assume the partial beam penetration through a jaw is same as through MLC leafs. Inadequacy of the current modeling of the rounded leaf ends in Eclipse using only two adjustable parameters (the DLG and the leaf transmission) is evident in Figs. 8(a) and 8(b). Upon improving in the modeling of the rounded leaf ends in the TPS, a better agreement should be expected independently at every MLC control point and at every point in the patient's body. This is because the ion chamber‐based optimization of the DLG allows accurate modeling of the dose only at a single point in the patient's body, while different values of the DLG may be required at other points in the body, in particular in regions of lower dose. Better modeling of the beam profiles for MLC‐delimited fields should lead to higher pass values in 3D gamma maps, which can be measured using ArcCHECK (Sun Nuclear; Melbourne, FL),^(27)^ or even 2D gamma maps in film measurements.

It is interesting that the DLG equal 2.0 mm provides a good fit in all sliding window tests. Resemblance of the leaf movement in IMRT plans to the movement in the sliding window test implies that IMRT plans probably do not require plan‐specific adjustments of the DLG, but we did not investigate IMRT plans here.

The value of 2.0 mm providing best fit in sliding window plans cannot be applied to the VMAT plans of brain with metastases that we investigated, where the optimum DLG was between 0.8±0.2 mm and 2.05±0.15 mm. We believe that the difference between the values of the DLG between IMRT and VMAT plans is caused by different contribution to the dose from the beam penumbra, which is a consequence of different patterns of leaf movement.

Although new TPS algorithms have become available in addition to AAA (e.g., Acuros XB; Transpire Inc., Gig Harbor, WA),^28^, ^29^ the concept of the DLG remains relevant for MLCs designed with rounded leaf ends. Only better modeling of the rounded leaf ends can address the problem by using more appropriate beam profiles in MLC‐delimited fields.

## V. CONCLUSIONS

This work is meant to enhance understanding of the concept of the DLG used in modeling the rounded leaf ends of MLC in VMAT plans, as well as to illustrate limitations of the model with a single‐value DLG. We have extended the meaning of the DLG from a single number to a function of two parameters: the distance (in the BEV) between the dose point and the leaf ending, and the width of the MLC slit (i.e., the position of the opposite leaf). We have described how variations of the plan‐specific values of the optimum (single‐value) DLG in VMAT plans may be linked to the DLG function, and be correlated with variations of leaf patterns during the dose delivery.

## ACKNOWLEDGMENTS

The authors would like to thank Robert Corns, Steven Thomas, Tim Fofonoff, Gustavo Fernandez, Claudia Mendez, Eric Harvey, and Sheryl Harrop for helpful discussions, and Tom Bryceland for assistance with phantom construction.
